# An Anti-Interrupted-Sampling Repeater Jamming Method Based on Simulated Annealing–2-Optimization Parallel Optimization of Waveforms and Fractional Domain Extraction

**DOI:** 10.3390/s25103000

**Published:** 2025-05-09

**Authors:** Ziming Yin, Pengcheng Guo, Yunyu Wei, Sizhe Gao, Jingjing Wang, Anxiang Xue, Kuo Wang

**Affiliations:** Xi’an Electronic Engineering Research Institute, Xi’an 710100, China

**Keywords:** anti-intermittent-sampling relaying jamming, simulated annealing–2-optimization parallel optimization, waveform design, fractional Fourier transform

## Abstract

Faced with increasingly complex electronic jamming environments, intra-pulse agility has become a primary method of anti-interrupted-sampling repeater jamming (ISRJ) for radar systems. However, existing intra-pulse agile signals suffer from high autocorrelation sidelobe levels and limited jamming suppression capabilities. These issues restrict the performance of intra-pulse agile signals in complex electromagnetic environments.This paper proposes an anti-interrupted-sampling repeater jamming method based on Simulated Annealing–2-optimization (SA-2opt) parallel optimization of waveforms and fractional domain extraction. Firstly, the proposed method employs the SA-2opt parallel optimization algorithm to optimize the joint frequency and chirp rate encoding waveform. Then, the received signal is subjected to the fractional Fourier transform (FrFT) and inverse transform to extract the target signal. Finally, jamming detection is conducted based on the multi-dimensional features of the pulse-compressed signal. After this detection, a time-domain filter is constructed to achieve jamming suppression. The optimized waveform exhibits the following advantages: the sub-pulses are orthogonal to each other, and autocorrelation sidelobe levels are as low as -20.7dB. The method proposed in this paper can achieve anti-ISRJ in the case of a high jamming-to-signal ratio (JSR). Simulation experiments validate both the effectiveness of the optimized waveform in achieving low autocorrelation sidelobes and the anti-ISRJ performance of the proposed method.

## 1. Introduction

As an important part of modern sensing system [1,2,3], radar ’s interference suppression ability determines the accurate acquisition of objects information in complex electromagnetic environment [4]. With the rapid development of electronic counter measure (ECM) technology, interrupted-sampling repeater jamming (ISRJ) has become one of the primary threats to the performance of modern radars due to its strong parameter adaptability, flexible jamming patterns, and high energy utilization [5,6]. The ECM jammer intercepts the radar’s transmitted signals, processes them with intermittent sampling, and uses a Digital Radio Frequency Memory (DRFM) to reconstruct and match the parameters of the jamming signals [7]. As a result, ISRJ can create highly realistic false objects or suppress real objects in the time–frequency domain [8,9,10,11]. In particular, in advanced radar systems such as phased-array radars and synthetic-aperture radars, ISRJ can effectively evade traditional anti-jamming measures through parameter optimization, leading to a significant increase in radar false alarm rates and a sharp decline in target detection probabilities, among other severe consequences [5,12,13,14]. Anti-ISRJ has become a core issue that urgently needs to be addressed in the field of radar jamming resistance [15,16]. In recent years, a series of advances have been made in the research on the mechanism and countermeasure strategy of ISRJ. Currently, there are two primary anti-ISRJ methods: the first involves anti-jamming measures solely at the receiver end, while the second employs joint anti-jamming strategies combining both transmitter and receiver. The first method focuses on extracting the target signal or suppressing the jamming signal at the receiving end through various transformations [17,18,19,20,21]. For instance, Liu et al. employed compressed sensing (CS) techniques to alter the sampling quantity of the received signal, reconstruct the target signal, and thereby suppress the jamming [17]. However, an anti-ISRJ method relying solely on the receiver often involves huge computation, making real-time jamming suppression challenging. The second method involves simultaneous suppression from both the transmitter and receiver. This is achieved by transmitting intra-pulse composite modulation signals, which divide a single pulse into multiple sub-pulses with different parameters. The mutual masking characteristics of these pulses are then utilized to detect and suppress jamming signals in the echo [12,22,23,24,25,26,27]. A notable example of this method is presented in [23], where Zhan et al. proposed orthogonal sub-pulse waveforms to create significant differences between the jamming and target signals in multiple dimensions, using the OTSU method for jamming suppression. However, changes in sub-pulse parameters can significantly affect the sidelobe peak of the entire pulse after pulse compression, leading to an increased signal-to-clutter ratio, which is detrimental to target detection. Additionally, multiple antennas can be used to transmit signals with different parameters. Chen et al. utilized the time–frequency differences in the received signals of distributed radars to reconstruct and suppress jamming signals by training a Minimum Variance Distortionless Response (MVDR) beamformer [28], but using multiple antennas can result in higher costs and limited application scenarios.

In response to above challenges, this paper adopts a joint suppression method at both the transmitter and receiver. The transmitted waveform uses a combined coding of sub-pulse carrier frequency and chirp rate, which confers some anti-interception characteristics. On this basis, the objective function is to minimize the sidelobe peak of the autocorrelation, and the Simulated Annealing–2-optimization (SA-2opt) parallel optimization method is used to optimize the transmitted waveform, achieving a sidelobe peak of −20.7 dB after waveform matching filtering, which is a reduction of approximately 10 dB compared to the unoptimized waveform, significantly enhancing the target detection performance. Furthermore, this paper employs the fractional Fourier transform (FrFT) to extract the target signal based on the different modulation rates of the transmitted waveform. The mean, variance, and histogram entropy of the segmented pulse compression results are then used as three-dimensional features and clustered using the K-means algorithm to construct a time-domain filter that further suppresses the jamming. The main contributions of this paper are summarized as follows:

1. Optimizing the radar signal coding, which involves alternating positive and negative modulation rates of sub-pulses and joint coding of modulation rate and carrier frequency. The problem is abstracted into a two-dimensional Traveling Salesman Problem (TSP), and an SA-2opt parallel optimization algorithm is proposed to find an approximate solution, ensuring good detection capabilities of the radar even in the absence of jamming.
2. Using segmented FrFT filtering and combining it with the K-means algorithm to complete ISRJ detection, achieving effective suppression of ISRJ in high-signal-to-clutter-ratio environments.

The rest of this article is organized as follows. Section 2 of this article establishes the ISRJ signal model and analyzes its countermeasures. Section 3 of this article provides a detailed exposition of the transmit waveform optimization method. Section 4 of this article proposes a joint jamming suppression algorithm. Section 5 of this article validates the effectiveness of the proposed scheme through simulations and real measurement data. Finally, Section 6 of this article summarizes the entire article.

## 2. Introduction of the Waveform Mathematical Model and Performance Analysis

### 2.1. Signal Model

Linear frequency-modulated (LFM) signals are used as sub-pulse waveforms, with different carrier frequencies and modulation rates set between sub-pulses. Specifically, if the transmitted signal consists of M sub-pulses, the *i* th sub-pulse $s_{sub}\left(t,i\right)$ can be expressed as

(1) $$s_{sub}\left(t,i\right)=u\left(t-i\tau_{sub}\right)\exp\left(j\pi \gamma_{sub}\left(i\right){\left(t-i\tau_{sub}\right)}^{2}\right)\exp\left(j2\pi f_{sub}\left(i\right)\left(t-i\tau_{sub}\right)\right)$$

where *t* is the fast time, $\tau_{sub}$ is the time width of the sub-pulse, $\gamma_{sub}\left(i\right)$ is the modulation rate of the *i* th sub-pulse, and $f_{\mathrm{sub}\;}\left(i\right)$ is the carrier frequency of the *i* th sub-pulse. u(t) is the window function and $u\left(t\right)=\left\{\begin{array}{cc} 1, & 0<t<\tau_{\mathrm{sub}\;}\\ 0, & \;\mathrm{other}\; \end{array}\right.$.

The transmitted signal can be expressed as

(2) $$s\left(t\right)=\sum_{i=1}^{M}s_{sub}\left(t,i\right)$$

The impulse response of the sub-matched filter can be expressed as

(3) $$h_{sub}\left(t,i\right)=s_{sub}^{*}\left(\tau -t,i\right)$$

**Figure 1:**
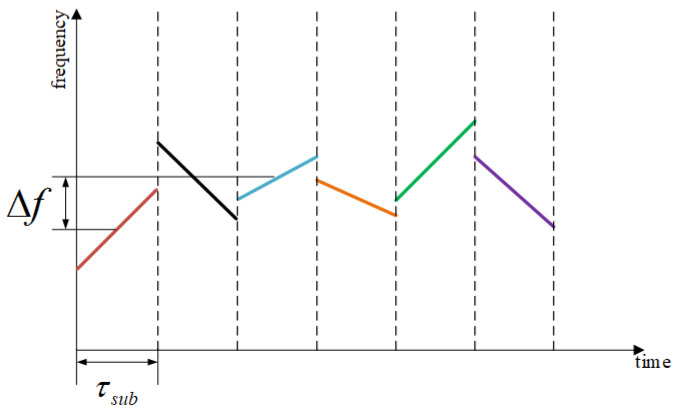
Time –frequency plot of the transmitted signal.

The transmitted signal can be expressed as

(4) $$\begin{array}{cc} s(t)= & \sum_{i=1}^{M}u\left(t-i\tau_{\mathrm{sub}\;}\right)\exp\left(j\pi \gamma_{\mathrm{sub}\;}\left(S_{r}\left(i\right)\right){\left(-1\right)}^{\left(i-1\right)}{\left(t-i\tau_{\mathrm{sub}\;}\right)}^{2}\right)\\ \; & \;\exp\left(j2\pi f_{\mathrm{sub}\;}\left(S_{f}\left(i\right)\right)\left(t-i\tau_{\mathrm{sub}\;}\right)\right)\exp\left(j2\pi \gamma_{\mathrm{sub}\;}\left(S_{r}\left(i\right)\right)\tau_{\mathrm{sub}\;}\left(t-i\tau_{\mathrm{sub}}\right)/2\right) \end{array}$$

where the frequency coding sequence of the sub-pulses is $S_{f}$ and the modulation coding sequence is $S_{r}$. $f_{sub}\left(\left(S_{r}\left(i\right)\right)\right)$ is the frequency of the *i* th sub-pulse and $\gamma_{sub}\left(\left(S_{r}\left(i\right)\right)\right)$ is the chirp rate of the *i* th sub-pulse. In order to enhance the difference in the chirp rate of adjacent sub-pulses, the chirp rate of adjacent sub-pulses is interleaved, which is expressed as the chirp rate $\gamma_{\mathrm{sub}}$ multiplied by ${\left(-1\right)}^{\left(i-1\right)}$. In Equation (4), the adjacent sub-pulses are connected end to end, and the sub-pulse delay and width are expressed by the window function as $u\left(t\right)$. The time–frequency plot of the transmitted signal s(t) is shown in Figure 1.

Δf is the minimum frequency hopping interval of sub-pulses, and the hopping interval of adjacent sub-pulses is an integer multiple of this value.

### 2.2. Analysis of the Necessity of Sub-Pulse Orthogonality

**Figure 2:**
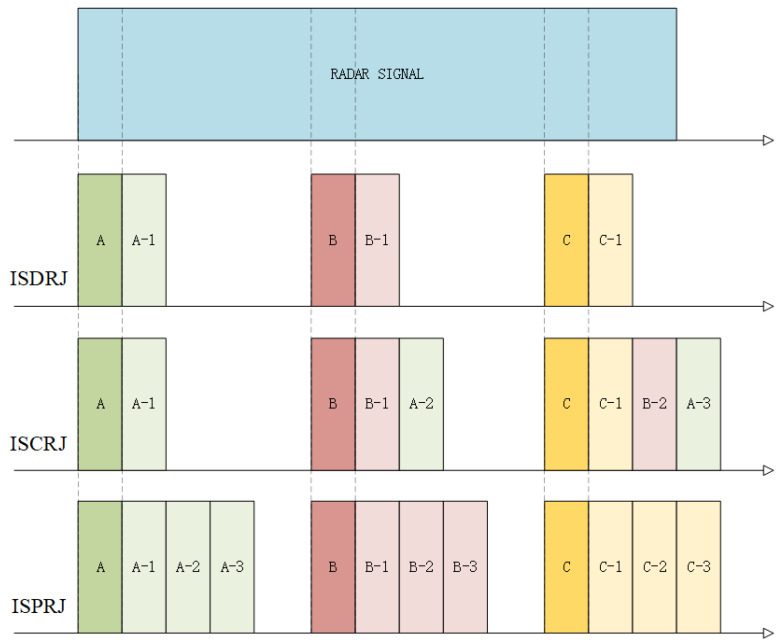
The operational mode of ISRJ.

The waveform design in Section 2.1 implements varying carrier frequencies and chirp rates across sub-pulses. Parameter diversity not only strengthens sub-pulse orthogonality but also amplifies inter-pulse distinctiveness, creating conditions for anti-ISRJ. ISRJ involves sampling and retransmission processes due to its generation mechanism. Intermittent-sampling repeater jamming usually uses two transmit–receive isolated antennas to work simultaneously to achieve fast reception and forwarding of signals, and sampling is performed first, followed by retransmission [18]. To enhance the jamming effect, the jammer should ensure that the entire sampled signal is retransmitted. Therefore, the minimum time width of the transmitted jamming signal is equal to the time width of the sampled signal. The operational mode of ISRJ is shown in Figure 2.

**Figure 3:**
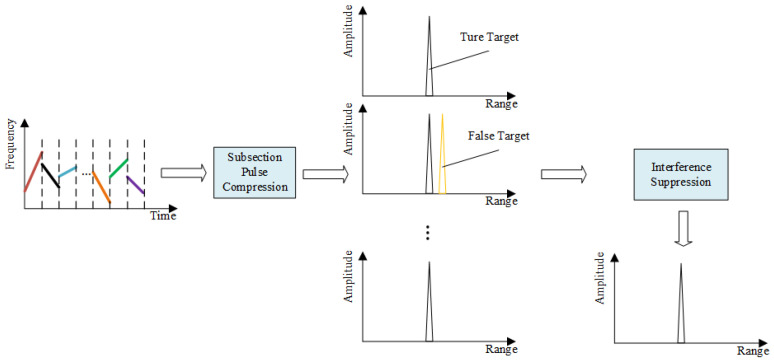
Jamming suppression process.

Depending on the retransmission method, ISRJ can be divided into Interrupted-Sampling Direct Repeater Jamming (ISDRJ), Interrupted-Sampling Periodic Repeater Jamming (ISPRJ), and Interrupted-Sampling Cyclic Repeater Jamming (ISCRJ). In Figure 2, the signals A, B, and C are the signal slices formed by the jammer’s sampling and copying of the radar signal. A-i represents the *i* th forwarding of signal slice A, B-i represents the *i* th forwarding of signal slice B, and C-i represents the *i* th forwarding of signal slice C. For ISRJ, the jamming energy is distributed within the current pulse. Therefore, the suppression of ISRJ must be completed within the current pulse. The ISRJ suppression strategy using intra-pulse agile waveforms employs a segmented matched filtering architecture. As shown in Figure 3, each sub-pulse undergoes dedicated filtering through corresponding sub-matched filters, followed by (a) jamming detection threshold analysis per filter channel and (b) selective accumulation of verified clean outputs.

Figure 3 illustrates the operational workflow of ISRJ countermeasures using intra-pulse agile signals. The results of segmented pulse compression in Figure 3 show that some sub-pulses contain jamming, and the jamming signal appears as an irregular peak after matched filtering, forming a false target in the radar receiver. The temporal lag between ISRJ signals and sampled counterparts ensures that during segmented pulse compression, each sub-matched filter remains optimally matched to the current sub-pulse. Consequently, jammer-retransmitted prior sub-pulses experience significant gain suppression in the current filter channel due to waveform mismatch. However, residual high-energy jamming components may still generate false targets at specific sub-pulse positions. Supplementary suppression techniques are needed to eliminate these artifacts.

Following segmented pulse compression of sub-pulses, the system requires jamming energy to be confined within the current segment without affecting others. Additionally, because the jamming signal is a complete replica of the prior sub-pulse, the effectiveness of jamming suppression will depend on the orthogonality between the sub-pulses. The orthogonality between sub-pulses can be expressed as the result of the inner product of two signals, as given by Equation (5).

(5) $$O\left(i_{1},i_{2}\right)=\frac{s_{sub}\left(t,i_{1}\right)\cdot s_{sub}\left(t,i_{2}\right)}{\left|s_{sub}\left(t,i_{1}\right)\right|\cdot \left|s_{sub}\left(t,i_{2}\right)\right|}$$

The value of Equation (5) being closer to 0 indicates stronger orthogonality between the two sub-pulses. The orthogonality of sub-pulses can also be intuitively reflected through their correlation. The weaker the correlation, the stronger the orthogonality. The cross-correlation between sub-pulses $i_{1}$ and $i_{2}$ can be expressed as

(6) $$R_{i1,i2}\left(\tau \right)=\int_{-\infty }^{+\infty }s_{sub}\left(t,i_{1}\right)s_{sub}\left(t+\tau ,i_{2}\right)dt$$

When τ=0, Equation (6) degenerates into Equation (5). When the two sub-pulses are not consistent in both carrier frequency and modulation rate, their cross-correlation function will not exhibit a significant peak at the center but will be relatively flat. Therefore, the orthogonality of sub-pulses directly affects the suppression of ISRJ, and the better the orthogonality, the less residual ISRJ energy there will be after suppression, resulting in better ISRJ suppression performance.

### 2.3. Analysis of Signal Autocorrelation Sidelobes

According to Section 2.2, improving the orthogonality of sub-pulses can enhance the radar’s capability for anti-ISRJ. However, focusing only on the orthogonality of the sub-pulses will lead to an increase in the autocorrelation sidelobe of the whole pulse. Specifically, the sidelobe peaks of the autocorrelation function of the entire pulse might become difficult to control due to the abrupt changes in frequency and modulation rate.

Based on the principle of matched filtering, the radar signal passing through the filter can be viewed as an autocorrelation process, and the peak of the autocorrelation sidelobes will affect the signal-to-clutter ratio. The sidelobe peaks of conventional LFM signals are inherently determined by the sinc function’s characteristic sidelobe structure. In contrast, the intra-pulse coded signal is composed of multiple LFM signals with different parameters, and the sidelobe peak changes according to the sub-pulse parameters. The autocorrelation function of the entire pulse can be expressed as

(7) $$\begin{array}{cc} R(\tau ) & =\int_{-\infty }^{+\infty }s\left(t\right)s\left(t+\tau \right)dt\\ \; & =\sum_{i=1}^{M}\sum_{j=1}^{M}\int_{-\infty }^{+\infty }s_{sub}\left(t,i\right)s_{sub}\left(t+\tau ,j\right)dt \end{array}$$

From Equation (7), it can also be seen that when the solution space does not change nonlinearly and continuously, the autocorrelation results of the signal have no obvious rules, and it is difficult to use the conventional method to solve the extreme value of the multivariate function. If the jamming signal has a certain correlation with some of the sub-matched filters, it will result a high peak after matched filtering.

When the sub-pulses are frequency-coded and chirp-rate-coded, and the positive and negative frequency modulation rates are alternated, both the frequency modulation rate and the hop frequency interval will affect the sidelobe peak of the entire pulse after matched filtering. As the sequence changes, the sidelobe peak variations do not follow a clear pattern. Without calculating the sidelobe peak, it is impossible to determine whether the sidelobe function value of a new sequence formed by swapping two elements in the original sequence will increase or decrease compared to the original sequence. Therefore, it is difficult to find low-sidelobe encoding sequences by analyzing the autocorrelation function of the waveform.

To find the encoding sequence with the lowest sidelobes, it is necessary to complete a search through all permutations, which is a typical Nondeterministic Polynomial (NP) problem. For example, with m sub-pulses, the search for the chirp rates of the sub-pulses requires *m*! operations, and the searching for the carrier frequencies of the sub-pulses also requires *m*! operations. The above two searches are combined with each other, so the search of the whole solution space needs to calculate ${\left(m!\right)}^{2}$ autocorrelation calculations. When m is large, the number of autocorrelation calculations will significantly increase, and the complexity of a single autocorrelation calculation is $O\left(N^{2}\right)$, where N is the number of sampling points in the entire pulse. The process of finding the sidelobe peak involves traversing the entire autocorrelation result; the computational complexity for determining the sidelobe peak of a single autocorrelation result is $O\left(N^{3}\right)$. When both m and N are large, traversing the entire solution space becomes extremely time-consuming, and a reasonable optimization algorithm should be used to complete the sequence search.

## 3. Introduction of Optimization Algorithm

### 3.1. Signal Sidelobe Optimization Model

The sidelobe peak of the radar echo signal after passing through the matched filter may degrade the SCR when spatially overlapped with clutter returns in the range–Doppler domain, whereas non-overlapping sidelobes primarily introduce ambiguities in multi-target scenarios rather than directly elevating the clutter floor. An excessively high sidelobe peak can even cause the radar to generate false targets. Minimizing the autocorrelation sidelobe peak is chosen as the objective function, with the constraint that the frequency modulation signs of adjacent sub-pulses in the time domain are opposite, and each element in the sequence appears only once. The optimization problem can be expressed as

(8) $$\begin{array}{cc} \; & \min\max R\left(\tau \right)=\int_{-\infty }^{\infty }s\left(t\right)s\left(t+\tau \right)dt\\ \; & \;s.t.\;\;\tau \in \{\;\mathrm{Excluding}\;\mathrm{the}\;\mathrm{main}\;\mathrm{lobe}\;\mathrm{area}\;\}\\ \; & S_{f}\left(i\right),S_{r}\left(i\right)\in \left\{1,2,\cdots ,m\right\},i=1,2,\cdots ,m \end{array}$$

After sampling, the autocorrelation function in the discrete state can be expressed as $x={\left[x_{1},x_{2},\cdots ,x_{N}\right]}^{T}$, and its corresponding matched filter is $h={\left[h_{1},h_{2},\cdots ,h_{N}\right]}^{T}$. Here, N is the length of the waveform and the corresponding matched filter after discretization. The radar pulse compression output R(k) can be represented as the cross-correlation of the two sequences.

(9) $$R\left(k\right)=\sum_{n=k+1}^{N}x\left(n\right)h^{*}\left(n-k\right),k=0,1,\cdots ,N-1$$

For convenience in representation, the process is described using matrix notation. Let the discrete signal matrix be X.

(10) $$X={\left[\begin{array}{ccccccc} x_{1} & x_{2} & \cdots & x_{N} & 0 & \cdots & 0\\ 0 & x_{1} & \cdots & x_{N-1} & x_{N} & \ddots & 0\\ \vdots & \ddots & \ddots & \vdots & \vdots & \ddots & 0\\ 0 & \cdots & 0 & 0 & x_{1} & \cdots & x_{N} \end{array}\right]}^{T}$$

In Equation (10), the matched filtering result can be expressed as R(k)=Xh.

To minimize the sidelobe peak during computation, the value at the main lobe position in the autocorrelation result can be set to zero, and then the maximum value in the sequence can be found as the sidelobe peak. This zero-setting operation can be achieved by multiplying by a weighted matrix W, i.e., W=diag{1,⋯,1,0,⋯,0,1,⋯,1}, where W is a diagonal matrix with the number of zeros in the middle equal to the width of the main lobe in the discrete autocorrelation function. Therefore, the radar signal sidelobe optimization problem can be abstracted as follows:

(11) $$\begin{array}{cc} \; & P\left(S_{f},S_{r}\right)=\min\max XhW\\ \; & \;s.t.\;S_{f}\left(i\right),S_{r}\left(i\right)\in \left\{1,2,\cdots ,m\right\},i=1,2,\cdots ,m \end{array}$$

### 3.2. Analysis of the SA-2opt Parallel Optimization Algorithm

From Section 2.3, the search for the entire sequence will result in significant time consumption, and a reasonable optimization algorithm needs to be adopted for the search. The SA algorithm is a heuristic global search algorithm that simulates the thermodynamic process of metal forging to obtain an approximate solution that tends toward the global optimum. The SA algorithm is widely used in both convex and non-convex optimization problems. The TSP is a classic NP-hard combinatorial optimization task that seeks the shortest closed tour visiting each given city exactly once and returning to the origin. The sub-pulse sequence optimization addressed in this paper, where each sub-pulse possesses distinct modulation parameters, can be formulated as a full permutation search across the parameter space. This problem is analogous to the TSP, where the complete route length (equivalent to the composite waveform’s peak sidelobe level, PSLL) can only be evaluated after finalizing the entire configuration. Therefore, the SA algorithm demonstrates superior applicability to sub-pulse sequencing problems. Alternative optimization methods, including Particle Swarm Optimization (PSO), Ant Colony Optimization (ACO), and Genetic Algorithms (GAs), exhibit limitations when addressing this specific optimization challenge.

**Figure 4:**
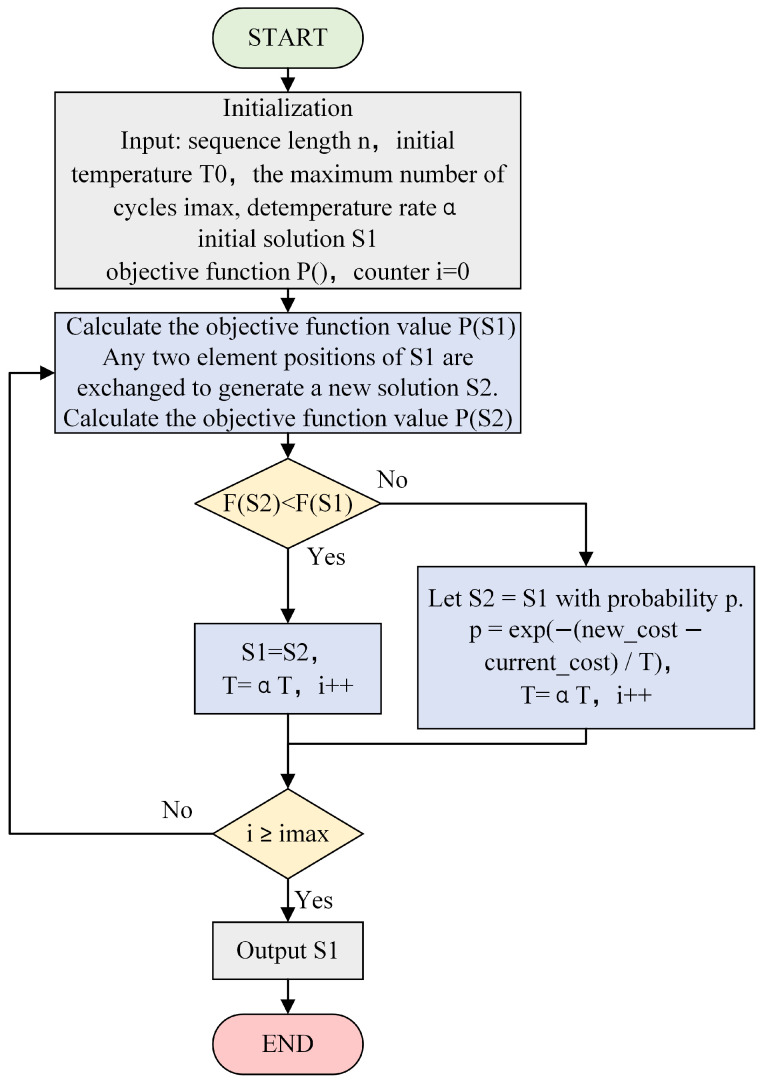
The SA algorithm flow.

When using the SA algorithm to solve the optimization coding problem, the objective function is to minimize the sidelobe level. On this basis, new solutions are generated by randomly producing new sequences. New solutions are accepted with a high probability if they are better, and the probability of accepting worse solutions is determined by the current temperature. The process continues to loop after accepting the new solution. The entire process of solving the optimization coding problem using the SA algorithm is shown in Figure 4.

Since the SA algorithm is primarily used to search for a one-dimensional optimal sequence, and the signal model in Section 2.1 involves a joint coding of two sequences, the problem to be addressed in this section is a two-dimensional optimal sequence search problem. Both the frequency coding and the chirp rate coding sequences simultaneously affect the objective function value. Therefore, when generating new solutions, two different sequences need to be generated simultaneously, and the methods for generating new solutions for these two sequences should differ to minimize the coupling phenomenon caused by the limitations of the solution generation methods, ensuring that the search can cover the entire solution space.

Moreover, to improve the search accuracy near the extremum points and to avoid potential coupling phenomena, the 2-opt algorithm is used to perform local searches based on the results of each SA iteration. When using the 2-opt algorithm for searching, the carrier frequency coding remains unchanged, while a one-dimensional search is performed on the chirp rate coding sequence to verify if there is a better chirp rate coding sequence under the same carrier frequency coding. The use of the 2-opt algorithm further reduces the coupling probability between the two coding sequences and avoids the optimization results falling into local optimum.

**Figure 5:**
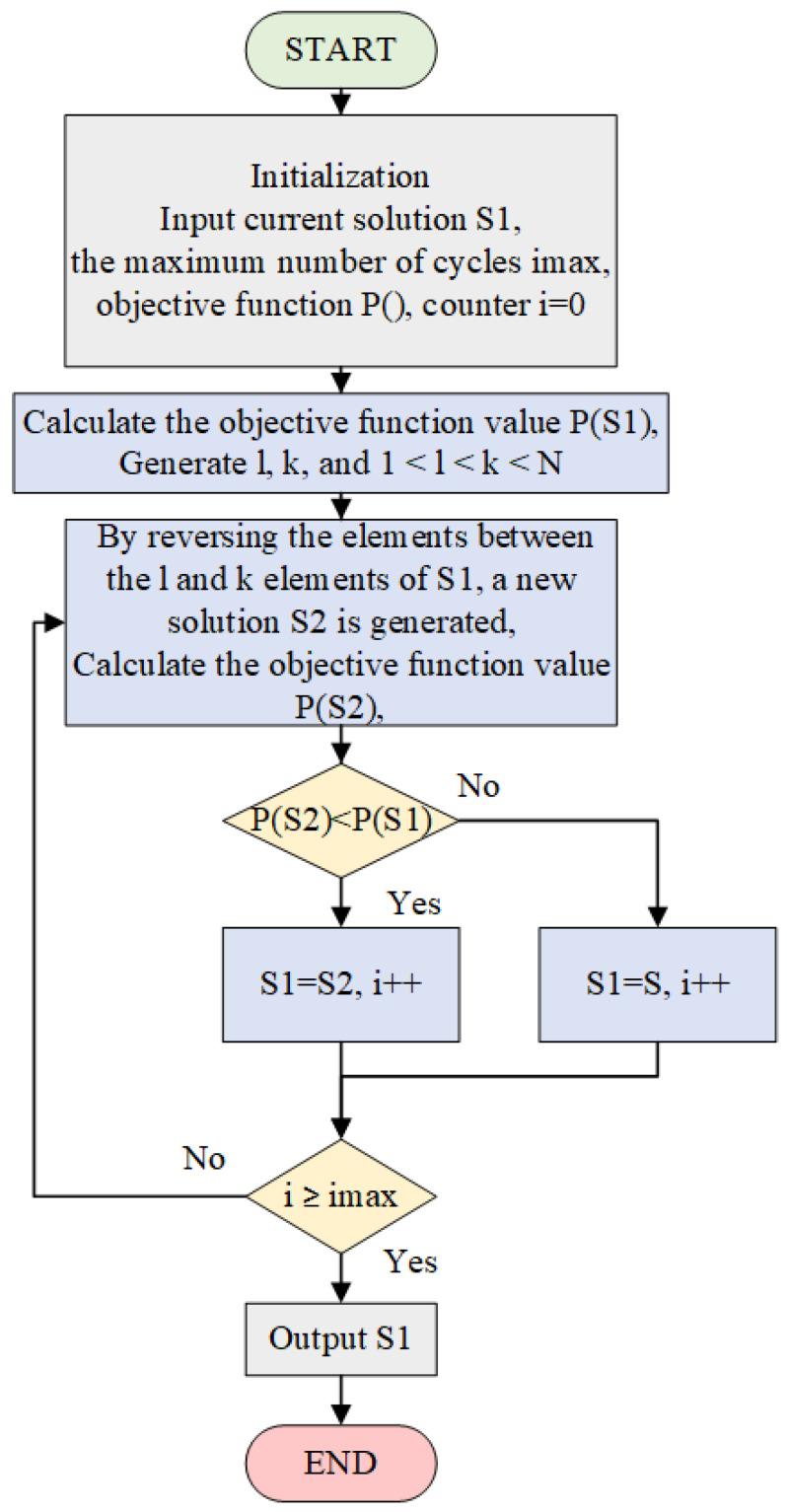
The 2-opt algorithm flow.

The 2-opt algorithm is a path improvement algorithm, which involves randomly generating two points and reversing the path segment between these two points, and then reinserting it into the original path to create a new path. If the objective function value decreases, the sequence is updated; otherwise, the process continues to loop until the maximum number of iterations is reached. The flowchart of the 2-opt algorithm is shown in Figure 5.

**Figure 6:**
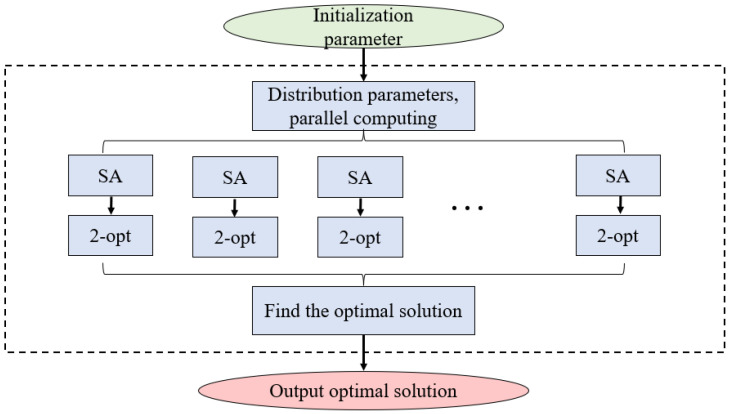
Flowchart of the SA-2opt parallel optimization algorithm.

To avoid the randomness introduced by the SA algorithm, multiple SA search processes are started in parallel, and the best solution among the parallel results is taken as the global approximate optimal solution. The algorithm structure and flow are shown in Figure 6.

In summary, this paper adopts a combination of the SA algorithm and the 2-opt algorithm, and performs multiple parallel Monte Carlo processes. The best solution from each process is selected. The specific steps are as follows:

**Step 1:** Initialize the algorithm parameters: initial temperature $T_{0}$ for the simulated annealing (SA) algorithm, cooling coefficient a, and the number of cooling repetitions $N_{1}$; the maximum number of iterations $N_{2\;\mathrm{opt}-\max\;}$ for the 2-opt algorithm; and the number of parallel processes $N_{par}$.

**Step 2:** Randomly generate $\;N_{par}\;$ initial sequence combinations $\left(S_{f}^{1},S_{mr}^{1}\right)$

$\left(S_{f}^{2},S_{mr}^{2}\right)L\left(S_{f}^{N_{par}},S_{mr}^{N_{par}}\right)$, each corresponding to one of the $N_{par}$ SA-2opt processes. For the k-th SA-2opt process, the steps are as follows:

(1) Calculate the objective function value $P\left(S_{f},S_{r}\right)$ at this point.

(2) Randomly generate two integers $k_{1}$ and $k_{2}$ between 1 and 8, and swap the elements of sequence $S_{f}^{k}$ at positions $k_{1}$ and $k_{2}$ to generate a new solution ${\left(S_{f}^{k}\right)}_{1}$. Similarly, randomly generate two integers $k_{3}$ and $k_{4}$ between 1 and 8, and swap the elements of sequence $S_{mr}^{k}$ at positions $k_{3}$ and $k_{4}$ to generate a new solution ${\left(S_{r}^{k}\right)}_{1}$.

(3) Set the carrier frequency $f_{1},f_{2},\cdots ,f_{m}$ to increment by Δf to obtain the carrier frequency vector *f*. Set the modulation frequency $r_{1},r_{2},\cdots ,r_{m}$ to increment by Δr to obtain the modulation frequency vector *r*.

(4) Sort *f* and *r* using sequences ${\left(S_{f}^{k}\right)}_{1}$ and ${\left(S_{r}^{k}\right)}_{1}$, and then compute the dot product of the sorted modulation frequency sequence *r* with ${\left(-1\right)}^{m}$ to finally calculate the objective function value $P\left({\left(S_{f}^{k}\right)}_{1},{\left(S_{r}^{k}\right)}_{1}\right)$.

(5) When $P\left({\left(S_{f}^{k}\right)}_{1},{\left(S_{r}^{k}\right)}_{1}\right)<P\left(S_{f},S_{r}\right)$, the current solution is updated to ${\left(S_{f}^{k}\right)}_{1},{\left(S_{r}^{k}\right)}_{1}$. When $P\left({\left(S_{f}^{k}\right)}_{1},{\left(S_{r}^{k}\right)}_{1}\right)>P\left(S_{f},S_{r}\right)$, accept $\left({\left(S_{f}^{k}\right)}_{1},{\left(S_{r}^{k}\right)}_{1}\right)$ as the new solution with probability $\exp\left\{-\frac{P\left({\left(S_{f}^{k}\right)}_{1},{\left(S_{r}^{k}\right)}_{1}\right)-P\left(S_{f},S_{r}\right)}{T_{0}\cdot a^{l}}\right\}$, where *l* is the current loop count.

(6) Denote the new solution as $\left(S_{f},S_{r}\right)$, and repeat steps (1) to (5) until $l>N_{1}$, at which point the loop terminates and $\left(S_{f},S_{r}\right)$ is output.

**Step 3:** The optimization results of **Step 2** need to be further optimized by the 2-opt algorithm. The specific steps are as follows:

(1) Generate an array ( i,j ) and select 1≤i<j≤m.

(2) Reverse the elements between positions i and j in the output of **Step 2** to generate ${\left(S_{r}\right)}_{2}$, and calculate the objective function value $P\left(S_{f},{\left(S_{r}\right)}_{2}\right)$. Compare $P\left(S_{f},{\left(S_{r}\right)}_{2}\right)$ with $P\left(S_{f},S_{r}\right)$ obtained from **Step 2**. If $P\left(S_{f},{\left(S_{r}\right)}_{2}\right)<P\left(S_{f},S_{r}\right)$, then update the solution to $\left(S_{f},{\left(S_{r}\right)}_{2}\right)$; otherwise, do not update.

(3) Repeat **Step 3** (1) and (2) until the number of iterations exceeds $N_{2opt-\max}$, and output the current solution denoted as ${\left(S_{f},S_{r}\right)}_{k}$, with the objective function value denoted as $P{\left(S_{f},S_{r}\right)}_{k}$.

**Step 4:** Select the solution ${\left(S_{f},S_{r}\right)}_{\min}$ corresponding to the minimum objective function value $P{\left(S_{f},S_{r}\right)}_{1}$ to $P{\left(S_{f},S_{r}\right)}_{N_{par}}$ from the entire process of 1 to $N_{par}$ as the final solution.

## 4. Anti-ISRJ Method

### 4.1. Echo Signal Model

After being affected by the ISRJ, the echo $S_{R}\left(t\right)$ will contain the target reflection signal $S_{T}\left(t\right)$, the jamming signal J(t), and the noise N(t), i.e.,

(12) $$S_{R}\left(t\right)=S_{T}\left(t\right)+J\left(t\right)+N\left(t\right)$$

Taking direct forwarding jamming as an example, in Equation (12), $S_{T}\left(t\right)=A_{1}S\left(t-\tau \right)$, $J\left(t\right)=A_{2}\sum_{j=0}^{M/2-1}u\left(\frac{t-\tau -\left(2j+1\right)\tau_{\mathrm{sub}\;}}{\tau_{\mathrm{sub}\;}}\right)S\left(t-\tau \right),A_{1}$ is the amplitude of the target signal, $A_{2}$ is the amplitude of the jamming signal at the radar receiver, and τ is the delay caused by the target distance.

### 4.2. Target Energy Extraction Based on the FrFT

According to Section 3.1, due to the use of carrier frequency and modulation rate coding, the carrier frequency and modulation rate of the target echo and the repeater jamming signal are different within the same sub-pulse width $\tau_{\mathrm{sub}\;}$. Transforming the received signal into the fractional domain can effectively distinguish the target signal from the jamming signal. The FrFT is an extension of the traditional Fourier transform, where the basis functions are expanded from single-frequency sinusoidal signals of the original Fourier transform to linear frequency modulation signals. The FrFT of the signal x(t) at angle α is given by

(13) $$X\left(u\right)=\int_{-\infty }^{+\infty }x\left(t\right)B\left(t,u\right)dt$$

where B(t,u) is the basis function of the transformation, and

(14) $$B\left(t,u\right)=\left\{\begin{array}{ccc} \sqrt{\left(1-j\cot\alpha \right)}\exp\left\{j\pi \left(t^{2}\cot\alpha -2ut\csc\alpha \right)+u^{2}\cot\alpha \right\} & , & \alpha \neq n\pi \\ \delta (t-u) & , & \alpha =2n\pi \\ \delta (t+u) & , & \alpha =(2n\pm 1)\pi \end{array}\right.$$

(15) $$p=\frac{\pi \alpha }{2}$$

where *p* is the order of transformation.

**Figure 7:**
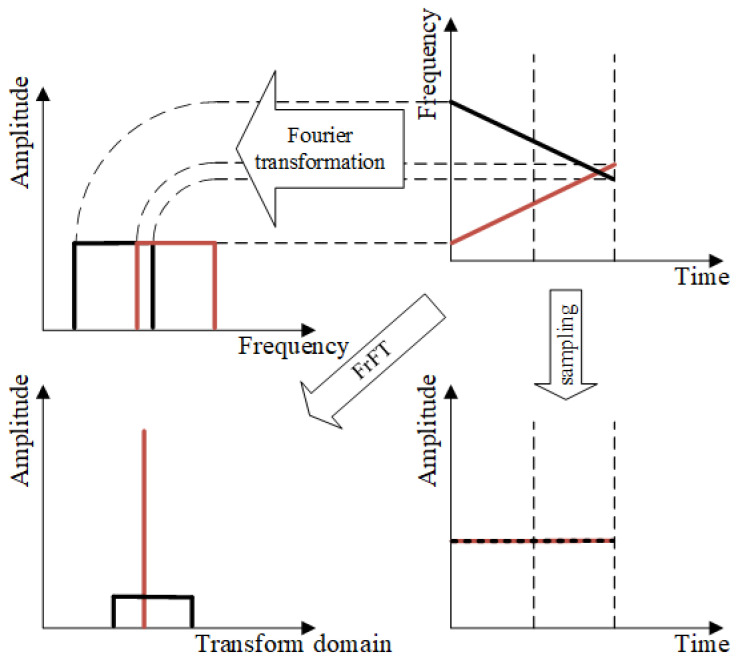
The FrFT schematic diagram.

The essence of rotating the time–frequency plane in the FrFT is to observe the time–frequency characteristics of the signal at different angles, providing a more flexible perspective for non-stationary signals such as LFM. The FrFT focuses on different positions based on the different chirp rates of LFM signals, projecting signals that are difficult to separate in the time domain or frequency domain onto different fractional domains to achieve separation. As shown in Figure 7, the time–frequency plot of the signal changes with the projection angle, resulting in different degrees and positions of concentration in the fractional domain.

By rotating the time–frequency plane, i.e., changing the value of α, the optimal projection angle for the LFM signals can be found, where the LFM signals show significant concentration. Therefore, to extract the LFM signal at this angle and minimize the mixing of other signals and jamming, it is necessary to find the rotation angle α that provides the best accumulation effect for the LFM signal. First, solve for the normalized chirp rate.

(16) $$k=\frac{B_{sub}}{f_{s}}$$

Then, α=arccot(−k) and $p=\frac{\pi }{2}\mathrm{arccot}\left(-k\right)$.

At this angle, the peak that appears is formed by the corresponding chirp rate signal. Since the transmitted signal in each sub-pulse has a clear difference in carrier frequency and chirp rate, the FrFT can be used to extract the target echo. The specific steps for target echo extraction are as follows:

**Step 1:** Divide the echo signal into *M* segments according to the number of sub-pulses, denoted as $S_{R}^{1},S_{R}^{2},\cdots ,S_{R}^{M}$.

**Step 2:** Use the corresponding FrFT angle based on the prior information of the signal parameters to transform the current segment of the signal into the fractional domain.

**Step 3:** Perform the fractional Fourier transform on the corresponding segments of the transmitted signal. For the k-th sub-pulse, construct a window function $u_{\mathrm{extract}\;}^{k}\left(x\right)=\left\{\begin{array}{cc} 1, & \;\mathrm{mainlobe}\;\\ 0, & \;\mathrm{others}\; \end{array}\right.$, where the main lobe region has a value of 1 and all other positions have a value of 0.

**Step 4:** Compute the inner product of $S_{R}^{k}$ and $u_{\mathrm{extract}\;}^{k}\left(x\right)$, which extracts the signal with the corresponding chirp rate, denoted as $S_{\mathrm{extract}\;}^{k}=S_{R}^{k}\cdot u_{\mathrm{extract}\;}^{k}\left(x\right)$. The jamming, due to its different chirp rate within the sub-pulse slice, cannot be effectively extracted.

**Step 5:** Perform an inverse transform on the retained main lobe to obtain the extracted target echo signal. The inverse transform is also a fractional Fourier transform, with the rotation angle being the reciprocal of the previous transformation angle, −α. Perform pulse compression on $S_{\mathrm{extract}\;}^{k}$ to obtain $s_{R}\left(t\right)$, which is the pulse compression result after extracting the target echo.

Through the above steps, the target echo within the signal slice can be partially extracted. However, during the target signal extraction process, some jamming signals still mix into the outputs. And the energy of the target signal outside the main lobe will be lost.

### 4.3. Segmented Pulse Compression and Jamming Suppression

The signal $S_{\mathrm{extract}\;}^{1},S_{\mathrm{extract}\;}^{2},\cdots ,S_{\mathrm{extract}\;}^{M}$ extracted in Section 4.2 is passed through sub-matching filters to obtain $S_{o}^{1}\left(t\right),S_{o}^{2}\left(t\right),\cdots ,S_{o}^{M}\left(t\right)$. $S_{\mathrm{extract}\;}^{1},S_{\mathrm{extract}\;}^{2},\cdots ,S_{\mathrm{extract}\;}^{M}$ contain target information, residual jamming, and noise. The outputs of each sub-matching filter are then accumulated. The residual jamming can still form multiple peaks near the accumulated target, so time-domain weighted filtering is required. To further suppress the jamming using time-domain weighted filtering, it is necessary to determine whether the post-slice signal contains jamming. Judging whether the echo signal is interfered with is difficult to achieve by setting a threshold for a single parameter. In interference detection, mean, variance, and entropy are multi-dimensional statistical features, which represent the concentration, discreteness, and randomness of signal energy distribution, respectively, and can jointly capture the non-stationary characteristics and statistical differences in interference signals in the time–frequency domain. By quantifying the statistical separability of interference and background noise, these features provide a robust criterion for various clustering methods, especially for blind detection and pattern recognition of non-cooperative interference in complex electromagnetic environments [29,30,31].

The mean value $\mathrm{mean}\left\{S_{O}^{k}\left(t\right)\right\}$ of the signal after matching filtering can reflect the intensity of the jamming. Since the matching filtering process produces significant peaks only at the positions of the target echo and jamming signals (including retransmitted jamming and strong noise jamming), the existence of the jamming signal significantly increases the mean value of the signal. The variance $\mathrm{var}\left\{S_{O}^{\kappa }\left(t\right)\right\}$ is also an important indicator for distinguishing whether the signal is jammed. Due to the presence of jamming, the matching filter output will form spikes at positions different from the real target, which will result in differences in the variance between the jammed and non-jammed signal slices after matching filtering. Additionally, information entropy can be used as a significant basis for determining whether the signal is interfered with. Information entropy is a fundamental concept in information theory and can effectively characterize the amount of information and the complexity of the signal. Some sub-pulses still contain jamming after segmented pulse compression, and the jamming signals can form spikes at corresponding positions after matching filtering, increasing the information content of the signal. Therefore, information entropy can be chosen as a feature for sub-pulse classification. Information entropy can be expressed as follows:

(17) $$H\left(X\right)=-\sum_{i=1}^{n}p\left(x_{i}\right)\log p\left(x_{i}\right)$$

where $p\left(x_{i}\right)$ is the probability of event $x_{i}$ occurring.

As a statistical measurement method, information entropy can be quantified by converting the signal into a histogram. In this paper, the output results of each matching filter are divided into 10 intervals based on their amplitude, and the ratio of the number of signal samples falling into each interval to the total number of samples is used as the probability in the histogram, thereby quantifying the signal to obtain the information entropy $H\left\{S_{o}^{k}\left(t\right)\right\}$ of each slice.

Therefore, this paper combines multiple features of the echo signal for determination, specifically the mean value, variance, and information entropy of the signal after matching filtering. The mean value, variance, and information entropy of the output results from each matching filter are arranged into a three-dimensional array $\;\left\{L_{1},L_{2},\cdots ,L_{M}\right\},\;$where $L_{k}=\left(\mathrm{mean}\left\{S_{o}^{k}\left(t\right)\right\},\mathrm{var}\left\{S_{o}^{k}\left(t\right)\right\},H\left\{S_{o}^{k}\left(t\right)\right\}\right)$. Since there is no prior information about whether the signal slice $S_{R}^{k}\left(t\right)$ is jammed, an unsupervised learning clustering method should be used to cluster $L_{k}$.

The K-means algorithm is one of the most widely used clustering algorithms today. It optimizes the within-cluster sum of squares iteratively to achieve data clustering. The core idea of K-means is to find the centroid of each cluster and assign data to the nearest cluster, continuously iterating to change the centroid positions so that the similarity between data points within the same cluster is maximized and the similarity between data points in different clusters is minimized. Using the K-means algorithm can fully leverage the information of the received signal across multiple dimensions, thus enabling jamming detection of sub-pulses.

In summary, the process of constructing the time-domain weighted filter can be divided into the following steps:

**Step 1:** Calculate the mean $\mathrm{mean}\left\{S_{o}^{k}\left(t\right)\right\}$, variance $\mathrm{var}\left\{S_{o}^{k}\left(t\right)\right\}$, and histogram information entropy $H\left(S_{o}^{\kappa }\left(t\right)\right)$ of each echo signal slice from Section 3.2, and map each slice to a three-dimensional point plot using these values as coordinates. The mean and variance calculations are not repeated here; the steps for calculating information entropy are as follows:

(1) Find the maximum value of $S_{o}^{k}\left(t\right)$ and normalize $S_{o}^{k}\left(t\right)$ to obtain $S_{o}^{\hat{k}}\left(t\right)=\frac{S_{o}^{k}\left(t\right)}{\max\left\{S_{o}^{k}\left(t\right)\right\}}$

(2) Divide the range 0∼1 into 10 intervals with a step of 0.1, and count the number of sample points of $S_{o}^{k}\left(t\right)$ falling into each interval, denoted as $D_{1},D_{2},\cdots ,D_{10}$.

(3) Calculate the histogram information entropy $H\left\{S_{o}^{\hbar }\left(t\right)\right\}=-\sum_{i=1}^{10}\frac{D_{i}}{n_{sub}}\log\frac{D_{i}}{n_{sub}}$, where $n_{\mathrm{sub}\;}$ is the number of sample points in $S_{o}^{k}\left(t\right)$.

**Step 2:** Use the K-means algorithm to classify the segment-mapped points obtained above, setting K=2, i.e., dividing them into two classes. The specific steps are as follows:

(1) Obtain the three-dimensional array $\left\{L_{1},L_{2},\cdots ,L_{M}\right\}$ from Step 1, where $L_{k}=(\mathrm{mean}\{S_{O}^{k}\left(t\right)\}),$

$\mathrm{var}\left\{S_{o}^{k}\left(t\right)\},H\left\{S_{o}^{k}\left(t\right)\right)\right\}$ is given initial centroids $c_{1}$ and $c_{2}$.

(2) For each point $L_{i}$, calculate its distance to the two centroids $d\left(L_{i},c_{1}\right)$ and $d\left(L_{i},c_{2}\right)$, and assign it to the cluster, $F_{1}$ or $F_{2}$, that contains the nearest centroid.

(3) Calculate the new centroid for each cluster: $c_{1}^{\prime }=\frac{1}{\left|F_{1}\right|}\sum_{x_{i}\in F_{1}}x_{i}$ and $c_{2}^{\prime }=\frac{1}{\left|F_{2}\right|}\sum_{x_{i}\in F_{2}}x_{i}$.

(4) If the update distance of all centroids is less than the threshold ϵ, stop the iteration; otherwise, repeat **Step 2** (1) to (3).

**Step 3:** Generally, it is believed that a slice is sampled and relayed in the next slice. Therefore, the first slice $S_{R}^{1}\left(t\right)$ is considered an uninterfered signal. The class containing $S_{R}^{1}\left(t\right)$ is defined as the uninterfered signal slice $G_{1}$, and the rest are defined as the interfered signal slices $G_{2}$.

**Step 4:** Pass the uninterfered slices through the matching filters constructed by the corresponding sub-pulses, and normalize the results to obtain the time-domain filter $H_{T}\left(t\right)$ and

(18) $$H_{T}\left(t\right)=\sum_{i\in G_{1}}S_{o}^{k}\left(t\right)$$

**Figure 8:**
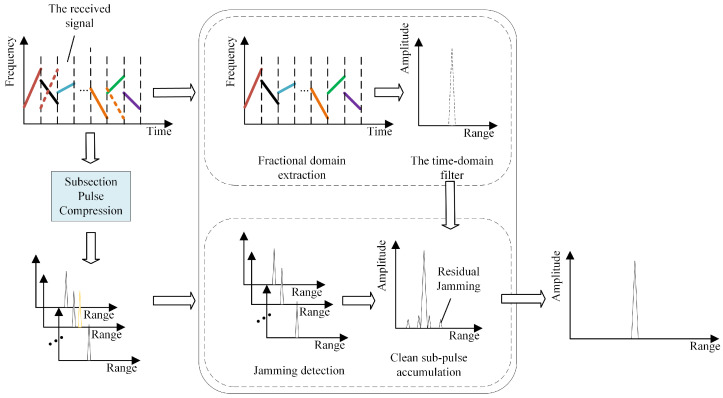
The interference suppression method in this paper.

Use the constructed time-domain weighted filter to weight $s_{R}\left(t\right)$. The target echo signal after jamming suppression is $s_{o}\left(t\right)=s_{R}\left(t\right)\cdot H_{T}\left(t\right)$. The overall jamming suppression process is shown in Figure 8.

## 5. Simulation Experiments and Analysis

In this section, four simulation experiments are established. Section 5.1 verifies the effectiveness of the optimized sequence sub-pulse coding waveform described in Section 3.2. Section 5.2 validates the stability and global performance of the method by comparing the results of traditional simulated annealing and 2-opt algorithms. Section 5.3 shows the performance of the optimized waveform when the target has radial velocity. Section 5.4 verifies the rationality of the algorithm parameter settings by changing the number of iterations. Section 5.5 validates the effectiveness of the optimized waveform and the jamming suppression measures described in Section 4.3 by comparing other jamming suppression methods. Section 5.6 discusses the influence of delay on jamming suppression.

### 5.1. Comparison of Sidelobe Peak Levels Before and After Optimization

**Figure 9:**
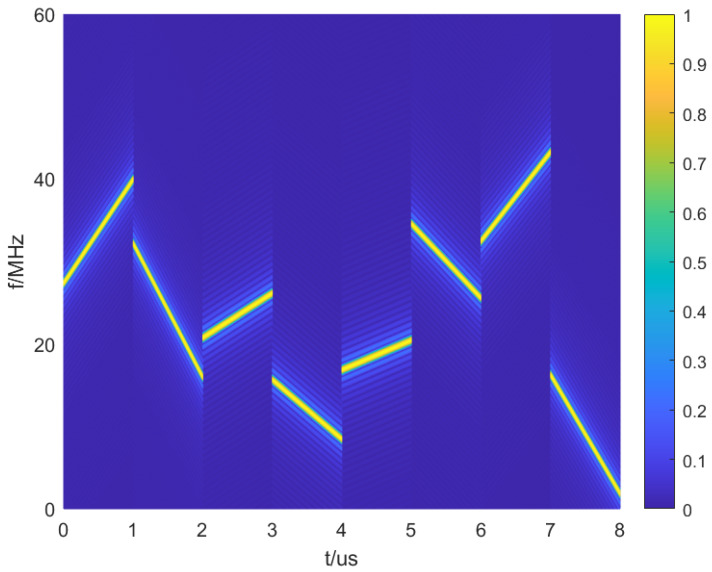
The time–frequency plot of the optimized waveform.

**Figure 10:**
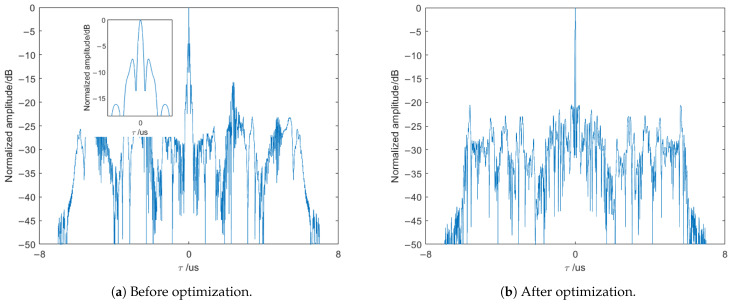
The autocorrelation function plot of the waveform.

The waveform parameters are set as shown in Table 1. Sequence optimization is performed under these parameters. The time–frequency plot of the optimized waveform is shown in Figure 9. Figure 10a shows the autocorrelation function plot of the waveform before optimization using a random sequence coding, while Figure 10b shows the autocorrelation function plot of the waveform after optimization using the optimized coding.

In Table 1, the minimum jump interval of the waveform is 3 MHz, and the jump interval of each adjacent sub-pulse is an integer multiple of this minimum interval. The absolute value of the frequency modulation of the waveform is selected as $8\times 10^{13}$×[0.2,0.3,0.4,0.5,0.6,0.7,0.8,0.9] Hz/s. On this basis, coding and frequency modulation positive and negative interleaving are performed. Since different coding will affect the synthesis bandwidth of sub-pulses, the maximum synthesis bandwidth of each sub-pulse is 50.6 MHz. According to the Nyquist sampling principle, the sampling rate needs to reach twice the bandwidth. At the same time, in order to avoid the influence of Doppler frequency caused by the target speed, the sampling rate is set to 160 MHz, which is higher than twice the maximum synthetic bandwidth.

From Figure 10a, it can be seen that when using a random sequence coding without optimization, the sidelobes of the waveform’s autocorrelation are relatively prominent, and in severe cases, they are only 7.8 dB below the mainlobe peak. In Figure 10b, after optimization using the SA-2opt parallel optimization algorithm, the autocorrelation sidelobes of the waveform are significantly suppressed, with the sidelobe peak reaching −20.7 dB. The sidelobe peak of the coding-modulated waveform after optimization is reduced by 12.9 dB compared to before optimization, which significantly suppresses the sidelobes and improves the signal-to-clutter ratio after radar matched filtering.

**Figure 11:**
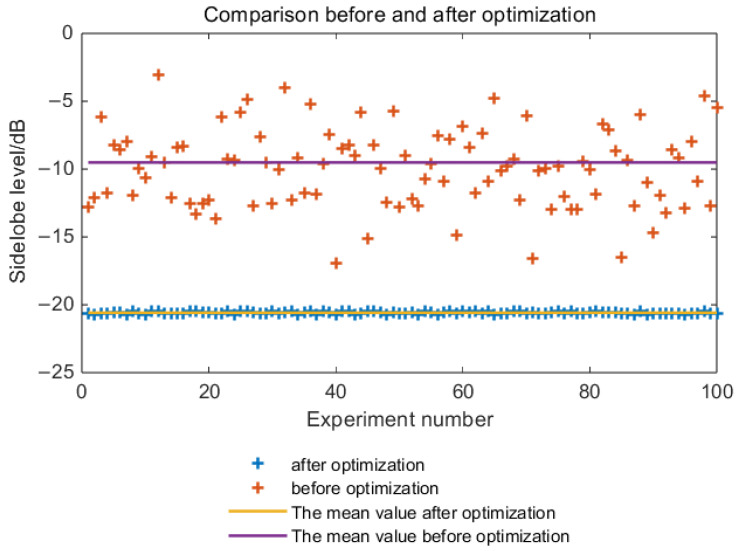
Monte Carlo experiment of sidelobe peak before and after optimization.

To more intuitively demonstrate the effectiveness of the optimized waveform, 100 Monte Carlo simulation experiments were conducted. The sidelobe peaks before and after optimization were calculated for each of the 100 simulations, as shown in Figure 11.

From Figure 11, it can be seen that the results of 100 Monte Carlo experiments and their statistical analysis indicate that the average sidelobe peaks before and after optimization are −10.2 dB and −20.6 dB, respectively. Before optimization, with random coding, the sidelobe peak fluctuates around −10.3 dB and can be as poor as −4.1 dB in some cases. Even under better conditions, it only reaches around −17.1 dB, making it difficult to directly apply for target detection. After optimization, the sidelobe peak is more stable, typically ranging from −20.3 dB to −20.9 dB, which significantly enhances the target detection performance.

### 5.2. Comparison with Traditional Optimization Algorithms

**Figure 12:**
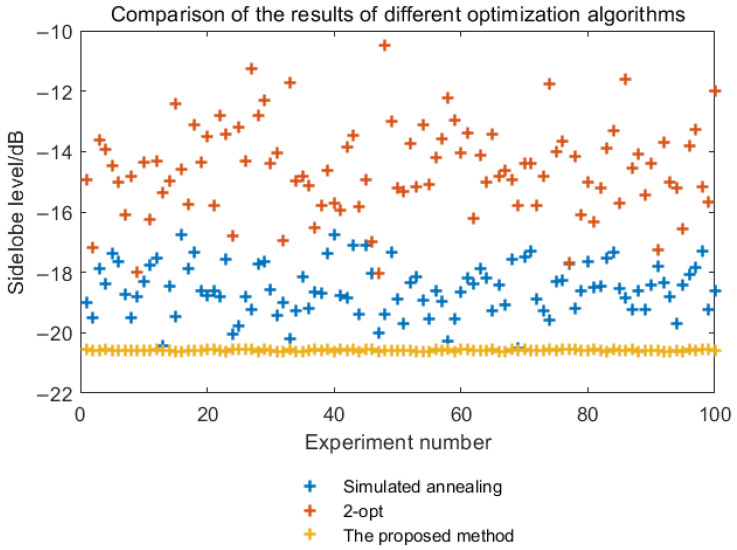
Comparison with traditional optimization algorithms.

Since this paper introduces the 2-opt algorithm and parallel Monte Carlo algorithm on the basis of the simulated annealing algorithm to enhance local search and global discovery performance, this will improve the stability of the search results. Figure 12 shows the comparison of search results between the proposed algorithm and traditional optimization algorithms (such as the simulated annealing algorithm and the 2-opt algorithm).

From Figure 12, it can be seen that the proposed algorithm outperforms the 2-opt algorithm and the simulated annealing algorithm in terms of result stability and optimization effectiveness. Due to the simultaneous and independent execution of multiple search processes in the proposed algorithm, it can search the entire solution space more effectively, leading to more stable results.

From Figure 12, it is evident that the search results of the simulated annealing algorithm fluctuate between −16.6 dB and −20.4 dB, while the sidelobe peak of the 2-opt algorithm ranges from −10.5 dB to −18.0 dB after optimization. In contrast, the sidelobe peak of the proposed SA-2opt parallel optimization algorithm is consistently between −20.5 dB and −20.7 dB. The above results indicate that the proposed algorithm outperforms the 2-opt algorithm and the simulated annealing algorithm in result stability and optimization effectiveness. The results show that the proposed algorithm outperforms the 2-opt algorithm and the simulated annealing algorithm in terms of stability and optimization effectiveness.

The reason is that the simulated annealing algorithm has a high degree of uncertainty in its search results and is prone to converging to local optimal solutions. Once it gets trapped in a local optimal solution, it struggles to escape, even with an increased number of subsequent iterations, leading to the waste of computational power and time. The 2-opt algorithm, on the other hand, is a local search algorithm and is generally used to find better solutions in the neighborhood of a feasible solution. It is not well suited for large-scale searches. The proposed algorithm can search the entire solution space more effectively, resulting in more stable and reliable results.

**Figure 13:**
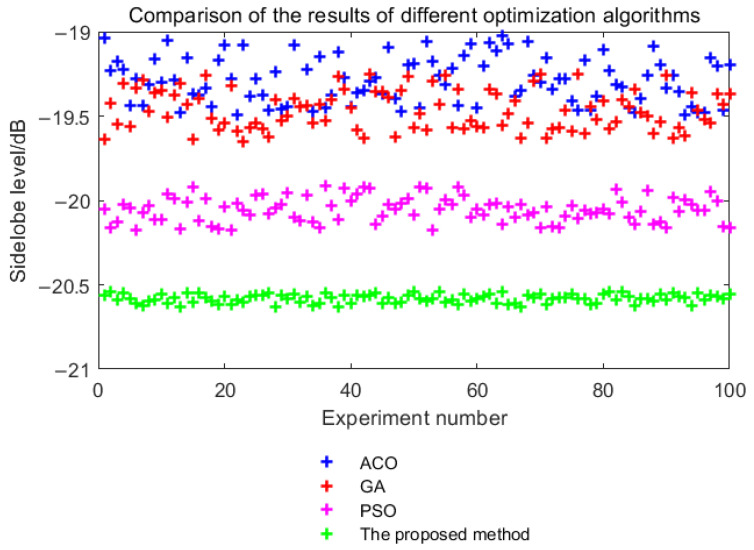
The comparison of the Monte Carlo optimization results.

**Figure 14:**
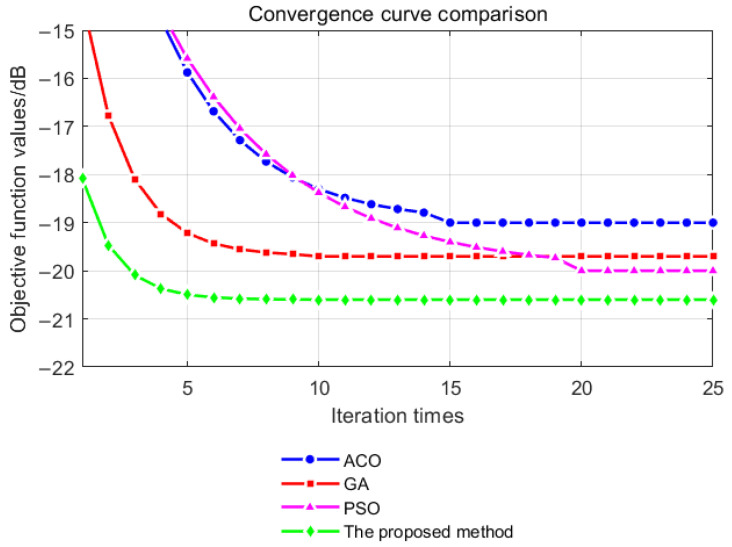
The comparison of the convergence curve.

In addition to the comparison with the SA algorithm and the 2-opt algorithm, comparison with other algorithms is also necessary. In this paper, the Ant Colony Optimization (ACO) algorithm, Genetic Algorithms (GAs), and Particle Swarm Optimization (PSO) algorithm are compared. The performance of the proposed algorithm and the above algorithms on the Section 3.1 optimization problem is compared, including the comparison of the Monte Carlo optimization results (Figure 13) and the convergence curve (Figure 14).

It can be seen from Figure 13 that the parallel optimization method used in this paper is more stable in the results and tends to the global optimal solution. It can be seen from Figure 14 that the convergence speed of the optimization algorithm in this paper is close to that of the PSO algorithm, which is better than that of the ACO algorithm and GA.

### 5.3. Performance of the Optimized Waveform When the Target Has Radial Velocity

**Figure 15:**
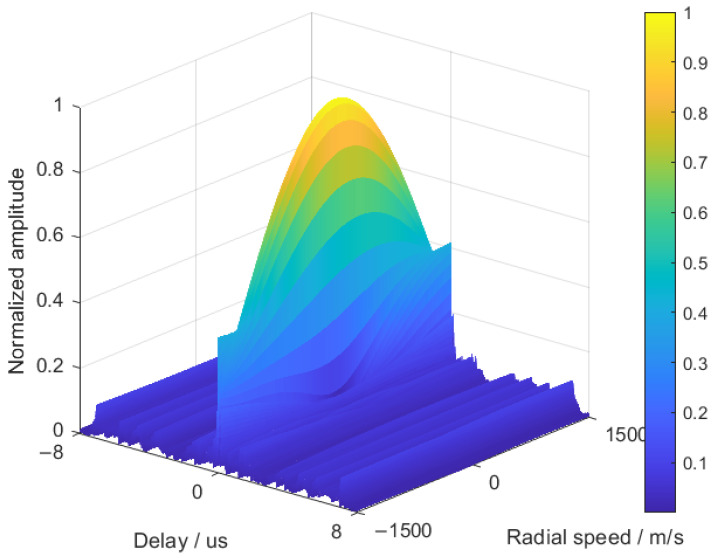
A part of the ambiguity function.

We further study the influence of Doppler frequency shift caused by target motion on the waveform in 5.1. Taking Ku band as an example, it is assumed that the carrier frequency is 10 GHz. According to the Doppler frequency shift formula of a radar system, when the target radial velocity is 100 m/s, the waveform in the XX section is adopted, and the sub-pulse frequency changes between 10.000 GHz and 10.041 GHz due to the joint agility of frequency and the frequency modulation slope. The Doppler frequency shift caused by the change in signal frequency and target velocity in the pulse will change between 0.00299 m/s and 0.00300 m/s, and the waveform detection performance will not decrease significantly. An ambiguity function is an effective tool for analyzing waveform performance. Figure 15 is the ambiguity function of the Section 5.1 waveform at the target speed of [−1500, 1500]. Table 2 shows the effect of target velocity on waveform pulse compression performance.

It can be seen from Table 2 that when the target velocity is within the range of (−500,500) m/s, the waveform designed in this paper has Doppler tolerance, and the pulse pressure loss is less than or equal to 1.04 dB. When the target speed is in the range of (−1500,−500)∪(500,1500) m/s, the pulse compression loss is less than or equal to 12.62 dB; when the target velocity is less than −1500 m/s or greater than 1500 m/s, the pulse compression loss is greater than 12.62 dB, which seriously deteriorates the signal-to-noise ratio of the target echo pulse compression.

In addition, the Doppler frequency shift will also affect the sub-pulse orthogonality of the target echo. Taking the speed of 100 m/s as an example, the carrier frequency of the whole pulse is 10.00 GHz, the maximum frequency interval of the sub-pulse is 24.00 MHz, and the maximum sub-pulse bandwidth is 16.20 MHz. The Doppler frequency shifts caused by the above factors are $6.67\times 10^{4}$ Hz, 16.00 Hz, and 10.80 Hz, respectively. It can be seen that the Doppler frequency shift of the target is reflected in the echo signal, which is more reflected in the frequency synchronization offset of the whole pulse. The frequency offset caused by the frequency jump of the sub-pulse and the target speed is not only far less than the frequency offset of the whole pulse, but also far less than the jump interval and bandwidth of the sub-pulse, which has little effect on the orthogonality of the sub-pulse.

### 5.4. Analysis of the Impact of Various Parameters on Optimization Results and Computation Time

For the proposed algorithm, the results and computation time are determined by two factors: the number of parallel Monte Carlo computations and the number of iterations in the simulated annealing algorithm. The hardware platform used in the simulation experiments of this section is configured with an Intel i5-13400F CPU, an NVIDIA GTX 1080Ti GPU, and 16G * 2 DDR5 4800 MHz memory. The software platform used is Matlab 2023a. Table 3 provides the runtime and optimized sidelobe peak values under different parameters:

From Table 3, it can be seen that the algorithm runtime increases almost linearly with the increase in the maximum number of iterations and the number of parallel computations. When the number of iterations is set to 2000 and the number of parallel computations is set to 30, a low sidelobe peak and a short runtime can be achieved, indicating that this state can already search for approximate solutions close to the optimal. The SA-2opt parallel optimization algorithm has low requirements for hardware platforms. Because of its parallel characteristics, the parallel processing ability of the hardware platform will directly determine the operation speed. The optimized waveform parameters can be written into the radar RF module, avoiding the time consumption of real-time calculations and making it feasible for engineering applications.

### 5.5. Waveform Anti-Jamming Performance

To reduce the waveform’s autocorrelation sidelobe peak, the coding sequence of the signal model described in Section 2.1 can be optimized using the SA-2opt parallel optimization algorithm. This optimization results in a transmitted waveform with a sidelobe peak of −20.7 dB, as shown in Figure 9.

**Figure 16:**
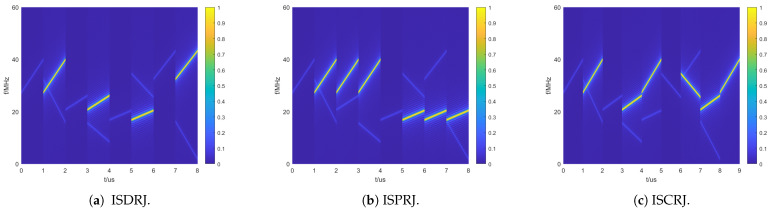
Time–frequency plots of three types of jamming.

The reception signal parameters are shown in Table 4. The following waveform anti-jamming experiments will be conducted based on these parameters. It is assumed that the jammer directly forwards the sampled signal without any delay, and the forwarded signal is identical to the sampled signal with no additional noise. The suppression effects of three types of jamming—ISDRJ, ISPRJ, and ISCRJ—are shown in Figure 16a–c. From left to right in the figure, the jamming types are ISDRJ, ISPRJ, and ISCRJ, respectively.

As shown in Figure 16a–c, in ISDRJ, the jamming signal is forwarded once at the start of the next sub-pulse after sampling the current sub-pulse. In ISPRJ, the jamming signal is forwarded three times at the start of the next sub-pulse after sampling the current sub-pulse. In ISCRJ, the jamming signal is forwarded in a cyclic manner, starting with the current sampled pulse, then the previous sampled pulse, and so on. The higher intensity stripes in the figure represent the jamming, i.e., ISRJ, while the darker stripes represent the target echoes.

**Figure 17:**
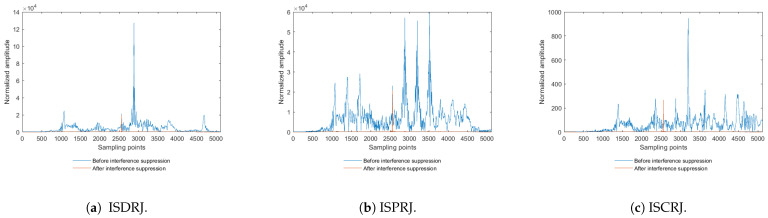
Comparison before and after jamming suppression for three types of jamming.

Following the general signal processing flow of a radar system (down conversion, pulse compression, and target detection), the range information of the target can be obtained after pulse compression. In the simulation experiment, the target is placed at the 2560th sampling point. The pulse compression results before and after applying the jamming suppression algorithm described in Section 4 of this paper are shown in Figure 17. Specifically, Figure 17a–c represent the pulse compression results under ISDRJ, ISPRJ, and ISCRJ, respectively.

In Figure 17, the amplitude of the false targets formed after pulse compression of the jamming signals is normalized based on the noise power. The ratio of the false target intensity to the true target intensity increases sequentially in Figure 17a–c. It can be observed that the number of false targets is determined by the number of times the sub-pulses are sampled and forwarded. Given a fixed jamming-to-signal ratio (JSR), the number of sampled sub-pulses determines the amplitude of the false targets. Before applying the jamming suppression measures, the true target is overwhelmed by the false targets, and in Figure 17a–c, the amplitude of the jamming signals’ sidelobes is even higher than the main lobe peak of the true target. After applying the jamming suppression measures described in Section 3.2, the false targets are largely suppressed, making the true target clearer. Additionally, the residual jamming can be suppressed using the time-domain filter.

**Figure 18:**
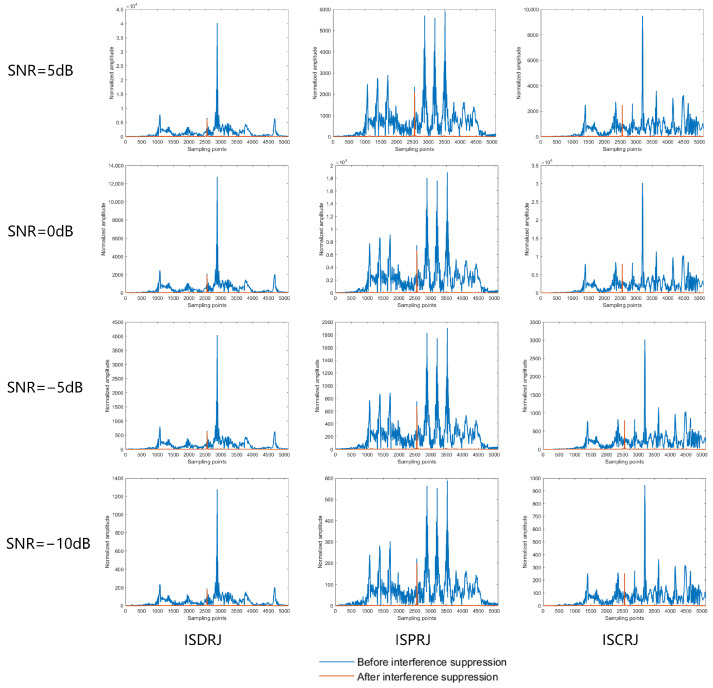
Anti-jamming performance under different signal-to-noise ratios.

To validate the environmental adaptability of the proposed method, we evaluate its anti-jamming performance between low-SNR (−10 dB) and high-SNR (10 dB) regimes. Figure 18 is the result of the interference suppression method in this paper at SNRs of 5, 0, −5, and −10.

It can be seen from Figure 17 and Figure 18 that the interference suppression method in this paper effectively suppresses the interference between −10 and 10 dB, which shows the robustness of the interference suppression method in a complex electromagnetic environment.

**Figure 19:**
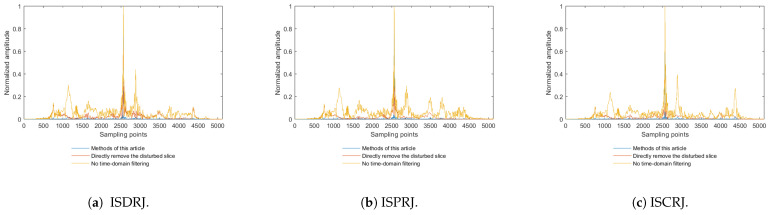
Comparison of jamming suppression results for ISDRJ, ISPRI, and ISCRJ.

Figure 19a–c show the comparison of the jamming suppression methods described in Section 4.3 with other methods, such as only removing sub-pulses affected by jamming or no time-domain filtering after pulse compression. From Figure 19a–c, it can be seen that directly discarding jammed sub-pulses would cause an amplitude loss in target signals after pulse compression. If applying pulse compression directly to FrFT-extracted target echoes, residual jamming persists, degrading subsequent detection performance. In contrast, the proposed method extracts target signals and integrates time-domain filtering post-compression, suppressing the residual jamming.

Since the entire pulse is divided into M sub-pulses, M segmented matching filters are required to process the target echo. Taking eight sub-pulses as an example, the running time of this process on MATLAB 2023 is 0.116412 s. The running time on Code Composer Studio 5.0 is 0.86 s. The above MATLAB 2023 operation results are based on a hardware platform with CPU i5-13400F and memory DDR5 4800 MHz. The running time result of Code Composer Studio 5.0 is based on a DSP TMS320C6678 chip. In addition, the interference suppression measures will also consume hardware resources equivalent to segmented pulse compression. Therefore, in practical engineering applications, signal processing chips with sufficient computing power are needed to ensure real-time performance.

### 5.6. Effect of Delay on Jamming Suppression

**Figure 20:**
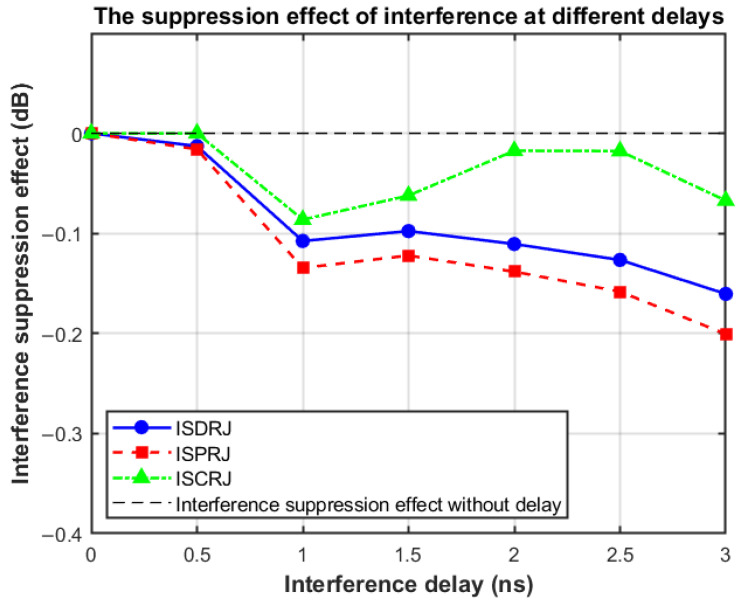
Anti-jamming performance under different delays.

In Section 5.5, we assume that the repeater of the jammer is not delayed, but this is different from the actual electronic warfare environment. In this section, we further evaluate the interference suppression effect of the proposed method after the interference signal is delayed by 0 to 3 ns (other simulation parameters remain consistent with Section 5.5) to simulate the radar signal forwarding by the conventional DRFM jammer. Normalization is based on the interference suppression effect when the delay is 0. The false target suppression effect corresponding to the interference delay is shown in Figure 20.

It can be seen from Figure 20 that the jamming forwarding delay of 0 to 3 ns basically does not affect the jamming suppression effect, and the proposed method can effectively achieve interference suppression. The performance change within 0.3 dB in Figure 20 also includes the performance loss caused by sampling, and the actual interference suppression effect should be less affected by the interference delay. As a comparison, the false target suppression effect is more than 40 dB when the delay is 0. Because the Doppler frequency shift caused by the target has little effect on the waveform performance, the combined effect of the interference delay and the target speed is also within the acceptable range within the interference delay of 0 to 3 ns. In addition, when the interference forwarding delay exceeds 400 ns, that is, 0.4 times the sub-pulse width, the accuracy of the K-means clustering algorithm will show obvious irregular changes. This is because the energy distribution of the interference signal is similar on the two adjacent sub-pulses of the target echo, so it is difficult to judge whether the sub-pulse is interfered. The correct judgment of whether the sub-pulse is disturbed will directly affect the interference suppression effect.

## 6. Summary

This paper focuses on intermittent-sampling forwarding jamming and optimizes the jointly coded intra-pulse frequency and chirp rate waveform to achieve the lowest autocorrelation sidelobe peak. The optimized waveform has the following advantages:

(a) The high degree of sub-pulse orthogonality effectively utilizes the mutual masking properties between sub-pulses, thereby establishing a clear discriminative boundary between the target echo and jamming signals.

(b) The low autocorrelation sidelobes, reaching −20.7 dB, enhance the signal-to-clutter ratio (SCR) after pulse compression, thereby improving target detection performance.

Additionally, this paper employs the FrFT to extract the target signal from receive signal and uses multi-dimensional features for interference detection based on the K-means algorithm. A time-domain filter is then constructed to suppress the jamming. Simulation experiments not only verify the low autocorrelation sidelobe characteristics of the optimized waveform, but also demonstrate the effectiveness of the proposed method in anti-ISRJ.

Adding detection probability (Pd) and false alarm probability (Pfa) is helpful for evaluating anti-jamming performance, but it is unreasonable to apply the above evaluation criteria to this article, because this article has used the pulse compression results after interference suppression to evaluate anti-jamming performance. In the future research, we will try to introduce pd and pfa to evaluate the anti-jamming performance. On the other hand, this paper has verified the effectiveness and robustness of the proposed method through parameter simulation experiments. In the future, the proposed method will be applied in engineering.

## Figures and Tables

**Table 1:** The waveform parameters.

Parameters	Values
Sub-pulse chirp rate (Hz/s)	$1.26\times 10^{13},-1.62\times 10^{13},5.4\times 10^{12},-7.2\times 10^{12},3.6\times 10^{12},$ $-9.0\times 10^{12},1.08\times 10^{13},-1.44\times 10^{13}$
Sub-pulse frequency hopping interval (MHz)	3
Sampling rate (MHz)	160
Sub-pulse width (μs)	1
Number of sub-pulses M	8

**Table 2:** The influence of target velocity on waveform performance.

Target Radial Velocity	Pulse Pressure Loss
0	0
±10 m/s	8.69 $\times \;10^{-4}$ dB
±100 m/s	4.00 $\times \;10^{-2}$ dB
±300 m/s	0.37 dB
±500 m/s	1.04 dB
±1000 m/s	4.55 dB
±1500 m/s	12.62 dB

**Table 3:** Runtime and optimized sidelobe peak values under different parameters.

Maximum Number of Iterations	Number of Parallel Computing	Running Time	Sidelobe Peak
5000	50	160 min	0.09
5000	10	32 min	0.10
2000	30	15 min	0.09
1500	100	40 min	0.09
1000	30	8 min	0.11
1000	100	60 min	0.10

**Table 4:** Received signal parameters.

Parameters	Values
Sub-pulse width (us)	1
Frequency hopping interval Δf (MHz)	3
Maximum bandwidth of sub-pulses (MHz)	16.2
Minimum bandwidth of sub-pulse (MHz)	3.6
Jamming-to-signal ratio JSR (dB)	20
Signal-to-noise ratio SNR (dB)	10

## Data Availability

The data are available to readers by contacting the corresponding author.

## DURC Statement

Current research is limited to the interference suppression in complex electromagnetic environment, which is beneficial for improving anti-jamming capabilities in radar systems (e.g., meteorological monitoring and navigation safety) and does not pose a threat to public health or national security. Authors acknowledge the dual use potential of the research involving waveform parameter optimization and confirm that all necessary precautions have been taken to prevent potential misuse. As an ethical responsibility, authors strictly adhere to relevant national and international laws about DURC. Authors advocate for responsible deployment, ethical considerations, regulatory compliance, and transparent reporting to mitigate misuse risks and foster beneficial outcomes.

## Abbreviations

The following abbreviations are used in this manuscript:

ECM	Electronic counter measure
DRFM	Digital Radio Frequency Memory
ISRJ	Interrupted-sampling repeater jamming
ISDRJ	Interrupted-Sampling Direct Repeater Jamming
ISPRJ	Interrupted-Sampling Periodic Repeater Jamming
ISCRJ	Interrupted-Sampling Cyclic Repeater Jamming
SA	Simulated annealing
2-opt	2-optimization
FrFT	Fractional Fourier transform
LFM	Linear frequency modulation
JSR	Jamming-to-signal ratio
SNR	Signal-to-noise ratio
